# Engineered Male Sterility by Early Anther Ablation Using the Pea Anther-Specific Promoter PsEND1

**DOI:** 10.3389/fpls.2019.00819

**Published:** 2019-06-25

**Authors:** Edelín Roque, Concepción Gómez-Mena, Rim Hamza, José Pío Beltrán, Luis A. Cañas

**Affiliations:** Department of Plant Development and Hormone Action, Biology and Biotechnology of Reproductive Development, Instituto de Biología Molecular y Celular de Plantas, CSIC-UPV, Valencia, Spain

**Keywords:** *barnase*, hybrid seeds, male sterility, parthenocarpy, *Pisum sativum*, pollen allergens, *PsEND1* promoter, transgene bioconfinement

## Abstract

Genetic engineered male sterility has different applications, ranging from hybrid seed production to bioconfinement of transgenes in genetic modified crops. The impact of this technology is currently patent in a wide range of crops, including legumes, which has helped to deal with the challenges of global food security. Production of engineered male sterile plants by expression of a ribonuclease gene under the control of an anther- or pollen-specific promoter has proven to be an efficient way to generate pollen-free elite cultivars. In the last years, we have been studying the genetic control of flower development in legumes and several genes that are specifically expressed in a determinate floral organ were identified. *Pisum sativum ENDOTHECIUM 1* (*PsEND1*) is a pea anther-specific gene displaying very early expression in the anther primordium cells. This expression pattern has been assessed in both model plants and crops (tomato, tobacco, oilseed rape, rice, wheat) using genetic constructs carrying the *PsEND1* promoter fused to the *uidA* reporter gene. This promoter fused to the *barnase* gene produces full anther ablation at early developmental stages, preventing the production of mature pollen grains in all plant species tested. Additional effects produced by the early anther ablation in the *PsEND1*::*barnase-barstar* plants, with interesting biotechnological applications, have also been described, such as redirection of resources to increase vegetative growth, reduction of the need for deadheading to extend the flowering period, or elimination of pollen allergens in ornamental plants (*Kalanchoe, Pelargonium*). Moreover, early anther ablation in transgenic *PsEND1::barnase-barstar* tomato plants promotes the developing of the ovaries into parthenocarpic fruits due to the absence of signals generated during the fertilization process and can be considered an efficient tool to promote fruit set and to produce seedless fruits. In legumes, the production of new hybrid cultivars will contribute to enhance yield and productivity by exploiting the hybrid vigor generated. The *PsEND1::barnase-barstar* construct could be also useful to generate parental lines in hybrid breeding approaches to produce new cultivars in different legume species.

## Introduction

Male sterility has been used by plant breeders to realize breakthroughs in the yield of different crops, through the development of hybrid cultivars. The impact of such technology is currently evident in some crops, including legumes (Saxena and Hingane, 2015), which has helped to deal with the challenges of global food security. Genes that are specifically expressed in the male reproductive organs could be used to obtain genetically engineered male sterile plants with potential applications in the production of hybrid seed, elimination of pollen allergens, or to avoid undesirable horizontal gene transfer in genetic modified (GM) crops.

Genetic cell ablation has been previously used to investigate male gametogenesis and as biotechnological tool to generate engineered male sterile plants using anther- or pollen-specific promoters fused to a cytotoxic gene (Koltunow et al., 1990; Mariani et al., 1990, 1992; Nasrallah et al., 1991; Paul et al., 1992; Dennis et al., 1993; Hird et al., 1993; Roberts et al., 1995; Zhan et al., 1996; Beals and Goldberg, 1997; De Block et al., 1997; Rosellini et al., 2001; Lee et al., 2003; Huang et al., 2016; Millwood et al., 2016; Yue et al., 2017). Production of engineered male sterile plants by expression of the ribonuclease *barnase* gene (Hartley, 1988), under the control of anther- or pollen-specific gene promoters, has been proved to be a good approach to generate pollen-free elite cultivars without adversely affecting the respective phenotypes (reviewed in Dutt et al., 2014; Mishra and Kumari, 2018). Moreover, male fertility can be restored in plants showing barnase-induced sterility by crossing with a transgenic line harboring the *barstar* gene, which encodes a powerful inhibitor of barnase (Mariani et al., 1992).

Genetic and molecular studies have revealed several important regulators of anther development, such as tapetum function, anther cell differentiation, or microspore development (Ma, 2005). Unfortunately, the expression of most of these genes was also observed in other floral or vegetative organs (Schiefthaler et al., 1999; Yang et al., 1999; Canales et al., 2002; Nonomura et al., 2003). However, *Pisum sativum ENDOTHECIUM 1* (*PsEND1*) is a pea anther-specific gene displaying very early expression in the anther primordium and along the anther development. The expression of this gene was not detected in other floral organs or vegetative tissues (Gómez et al., 2004). Therefore, due to their specific temporal and spatial expression pattern, the promoter of *PsEND1* was considered a useful tool to produce male sterile plants (Roque et al., 2007).

## *PsEND1* an Early Expression Anther-Specific Gene of Unknown Function

The PsEND1 protein was identified by our group several years ago following an immunosubtractive approach (Cañas et al., 2002). We were able to produce a series of monoclonal antibodies which specifically recognize proteins only present in a determinate floral organ. One of these antibodies recognized a protein of 25.7 kDa that was only detected in stamen extracts but not in the other floral organs, seeds, or vegetative tissues. The PsEND1-sequenced peptide presented a 79.3% identity with the N-terminus of the pea albumin PA2 (M17147; UniProtKB-P08688), which is only detected in the cytosol of cotyledonary cells (Harris and Croy, 1985; Higgins et al., 1987; Vigeoles et al., 2008). To isolate the *PsEND1* gene (GenBank AY091466) the similarity between the PsEND1 and PA2 proteins was very useful (Gómez et al., 2004).

**FIGURE 1:**
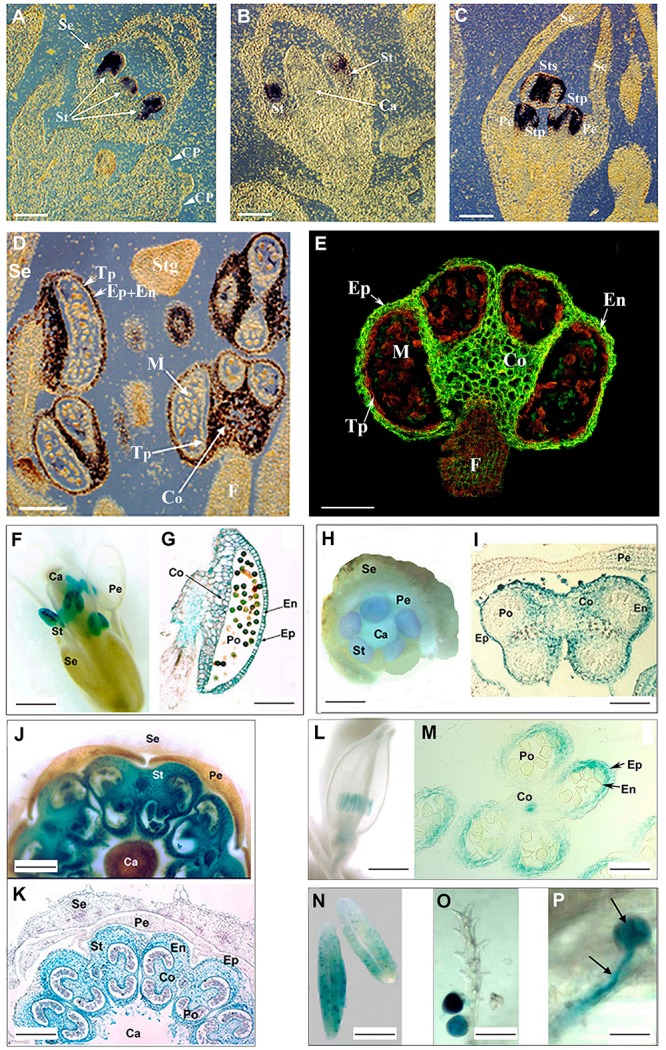
*PsEND1* expression in pea and other plant species. **(A)** RNA *in situ* hybridization in sections of two pea floral buds using digoxigenin-labeled antisense *PsEND1* RNA probes. Purple color indicates the localization of *PsEND1* expression. No expression was detected in the common primordia (CP) to petals and stamens (white arrows). The expression of *PsEND1* begins to be detected in the stamen primordia (St) of floral buds at day 12 before anthesis (d-12). **(B)** In flowers at d-10, the *PsEND1*expression is detected in the upper part of the stamen primordia where the anther locules will develop. **(C)** In flowers at d-8, *PsEND1* expression is only detected in those tissues that will be involved in anther architecture both in antesepalous and antepetalous stamens (Sts, Stp). **(D)** Close-view of a flower at d-6 showing anthers with strong hybridization signal in the epidermis (Ep), endothecium (En), middle layer, and connective (Co). No expression was detected in the anther filament (F), tapetum (Tp), and microspores (M). **(E)** Immunolocalization (anti-IgG-FITC) of the PsEND1 protein in paraffin sections of a pea stamen. The protein is localized (green fluorescence) in the same anther tissues than the RNA. **(F)** *PsEND1*::*uidA* expression in transgenic *Arabidopsis thaliana* flowers. GUS activity (blue) was only detected in the anther but not in the filament. **(G)** Transgenic *A. thaliana* anther section showing GUS activity in the structural tissues of the pollen sacs. **(H)** Young *PsEND1*::*uidA Nicotiana tabacum* flower showing GUS activity only in the stamen (St) primordia. **(I)** Transgenic *N. tabacum* anther showing GUS activity in the structural tissues of the pollen sacs but not in the pollen grains or tapetum. **(J)** *Solanum lycopersicum PsEND1*::*uidA* flower showing specific GUS activity in the anthers. **(K)** Transgenic tomato flower section showing GUS activity in the tissues involved in the architecture of the pollen sacs but not in the tapetum or in the pollen grains. **(L)** Expression of the *PsEND1*::*uidA* construct in the anthers of an *Oryza sativa* floret. **(M)** Section of a rice floret showing GUS activity in the expected anther tissues. **(N)** Expression of the *uidA* gene in the anthers of transgenic *Triticum aestivum* plants carrying the *PsEND1*::*uidA* construct. **(O)** Mature pollen adhering to the stigma showing GUS activity in a transgenic wheat flower. **(P)** Close-view of a germinating pollen grain, with pollen tube (arrows) growing in the style. Ca, carpel; Co, connective; En, endothecium; Ep, epidermis; Pe, petals; Po, pollen; Se, sepals; St, stamens; Tp, tapetum. Scale bars represent 100 μm in **A**, **B**, **C**, **D**, **E**, **G**, **I**, **K**, and **M**; 2.0 mm in **F**, **H**, **J**, and **L**; 0.5 mm in **N**; and 200 μm in **O** and **P**. Adapted from Gómez et al. (2004), Roque et al. (2007), Beltrán et al. (2007), and Pistón et al. (2008).

The anther-specific expression of *PsEND1* was elucidated by means of Northern blot and RNA *in situ* hybridization analyses (Gómez et al., 2004). The *PsEND1* expression pattern along stamen development demonstrated that this gene is active in the anthers from very early stages to 1 day (d-1) before anthesis. *In situ* hybridization assays showed that *PsEND1* expression begins in the stamen primordium, just in the moment when the common primordia (Benlloch et al., 2003) differentiate into petal and stamen primordia (Figure 1A). At late stages, *PsEND1* expression was detected in the epidermis, connective, middle layer, and endothecium, but not in the tapetum and microspores (Figures 1B–D). The PsEND1 protein was detected by immunolocalization in the same anther tissues (Figure 1E) and localized in the cytosol (Gómez et al., 2004). Due to the lack of efficient protocols for pea transformation, the function of *PsEND1* is to date unknown. The PsEND1 protein shows four copies of a hemopexin-type conserved repeat (Beltrán et al., 2007). Therefore, PsEND1 is related structurally to a group of mammalian regulatory proteins, in which the vitronectin is included (Jenne, 1991). The biological function of PA2 is still unclear because it does not present the classic features of a storage protein: PA2 lacks a signal peptide and it is not degraded during germination (Higgins et al., 1987). PA2 could play a role in controlling biological processes as a regulatory protein, dependent on ligand availability (Pedroche et al., 2005; Vigeoles et al., 2008).

## The Pea *PsEND1* Promoter is Functional in a Wide Number of Dicot and Monocot Species

The specific and early expression pattern of *PsEND1* suggested that the isolation of its promoter region would be of significant relevance to produce engineered male sterility. After screening of a genomic DNA library of pea and sequencing, a fragment of 2,946 bp was subcloned (GenBank AY324651). To assess whether the isolated *PsEND1* promoter sequence can specifically direct the expression of a foreign gene to the anthers of plants other than pea we transformed *Arabidopsis*, tobacco, oilseed rape, and tomato plants with a 2,731 bp fragment of the promoter sequence fused to the coding sequence of the *uidA* reporter gene (Gómez et al., 2004). The expression of the reporter gene was subsequently observed by histochemical analyses of GUS activity in seedlings, stems, leaves, roots, and flowers of kanamycin-resistant plants. Our results showed that the *PsEND1* promoter sequence was fully functional in all plant species tested. GUS activity was only observed in anthers, in the same tissues than pea, from very early stages of development to dehiscence (Figures 1F–K).

Alternatively, we have also assayed the *PsEND1*::*uidA* construct in two monocots: rice and wheat (Beltrán et al., 2007; Pistón et al., 2008). In transgenic rice (*Oryza sativa*) carrying this construct, GUS activity was detected in the same anther tissues in which the *PsEND1* expression has been previously described and, additionally, in the floret receptacle (Figures 1L,M). In transgenic wheat (*Triticum aestivum*) lines, GUS activity was firstly observed along pollen development, in the microspores at binucleate stage. *uidA* gene expression was also detected in mature pollen grains after anthesis. After pollen grain germination, *uidA* expression was seen from early (stigma attachment) to advanced stages (style progression) of pollen tube development (Figures 1N–P). No further GUS activity was detected after fertilization and during seed development (Pistón et al., 2008).

## Engineered Male Sterility in Model and Crop Plants Using the *PsEND1*::*Barnase-Barstar* System

A chimeric construct was generated joining the 2,731 bp fragment of the *PsEND1* promoter sequence to the *barnase* gene, which encodes a non-specific and very active ribonuclease. To prevent the undesirable effects of a possible ectopic expression of this gene, Gardner et al. (2009) proposed its use in combination with the *barstar* gene, thus protecting against the inappropriate expression of this active ribonuclease.

**FIGURE 2:**
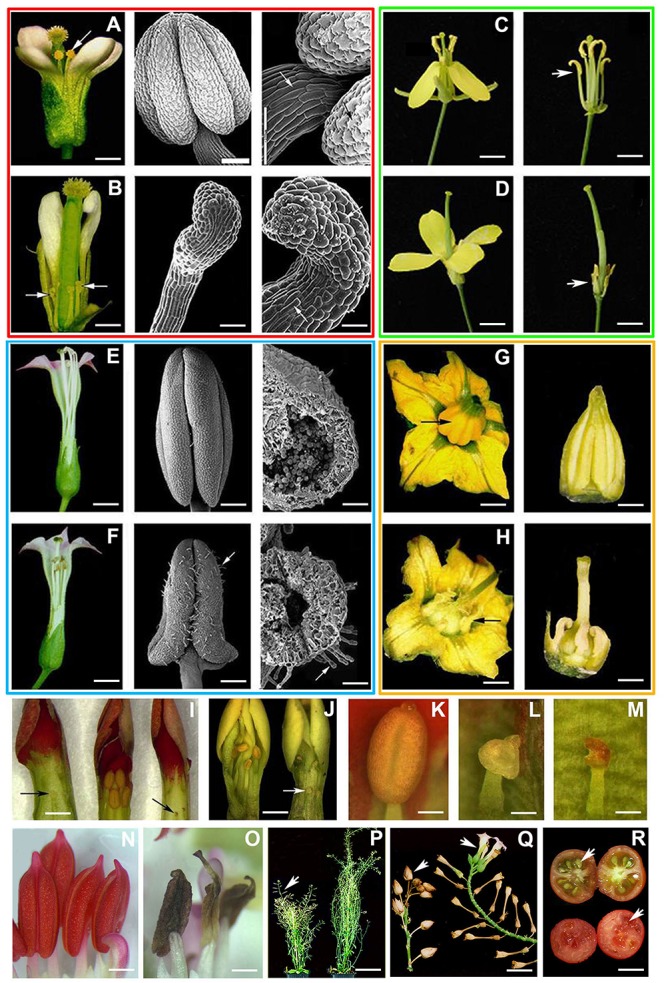
Engineered anther ablation in model plants and crops. Red box (*A. thaliana*). **(A)** Left: wild-type (WT) *A. thaliana* flower showing normal anthers (arrow). Center and right: WT *A. thaliana* stamen observed by scanning electron microscopy (SEM). The black arrow indicates the cell types (toothed edges) present in the anther epidermis and the white one those of the filament (lengthened). **(B)** Left: transgenic *A. thaliana PsEND1*::*barnase-barstar* flower (two sepals and two petals were detached). Anther ablation is evident and no pollen sacs were formed (white arrows). The anther filament is short because it does not undergo the lengthening process. Center and right: *PsEND1*::*barnase-barstar* stamen observed by SEM. The hook-shaped structures (white arrows) shown are cellular types usually present in the filament but not those present in the epidermis of WT pollen sacs. Green box (*B. napus*). **(C)** Left: WT oilseed rape (*Brassica napus*) cv. Drakkar flower showing normal stamens. Right: Id, but with detached sepals and petals to observe the normal anthers and filaments (white arrow). **(D)** Left: male sterile flower of a *PsEND1::barnase-barstar* oilseed rape plant showing the absence of developed stamens. Right: Id, but with detached sepals and petals to see the ablated anthers and the reduction of the filament length (white arrow). Blue box (*N. tabacum*). **(E)** Left: WT tobacco (*N. tabacum*) cv. Petite Havana SR1 flower after anthesis showing normal anthers and full-length filaments. Center: WT tobacco anther with its characteristic four locules fully developed observed by SEM. Right: section of a WT tobacco pollen sac showing mature pollen grains. **(F)** Left: *PsEND1*::*barnase-barstar* tobacco flower after anthesis showing collapsed lobes and reduced filaments. Center: Tobacco *PsEND1*::*barnase-barstar* anther showing an arrowhead shape with collapsed locules and increased number of trichomes (white arrow). Right: section of a *PsEND1*::b*arnase-barstar* pollen sac, no pollen grains can be observed into the collapsed locules. Orange box (*S. lycopersicum*). **(G)** Left: WT tomato (*S. lycopersicum*) cv. Micro-Tom flower at anthesis. Showing the staminal cone (black arrow) formed by the fully developed stamens in the center. Right: Isolated WT staminal cone covering the carpel. **(H)** Left: tomato *PsEND1*::*barnase-barstar* flower at anthesis. Right: anther ablation in the *PsEND1*::*barnase-barstar* flowers made visible the style and ovary of the carpel. **(I)** Flowers from a *Kalanchoe blossfeldiana* cv. “Tenorio” WT plant (center) and two male sterile lines (left and right) 1 day prior to anthesis. The WT flowers show anthers with fully developed locules, whereas the transgenic ones show collapsed structures at the end of a short filament instead of a four-lobed anther (black arrows). **(J)** Flowers from a *K. blossfeldiana* cv. “Hillary” WT plant (left) and a male sterile line (right) 1 day prior to anthesis with ablated anthers (white arrow). **(K)** WT anther from a “Tenorio” plant showing the normal four-lobed shape. **(L)** Close-view of a *PsEND1*::b*arnase-barstar* “Tenorio” ablated anther with a short filament. **(M)** Close-view of a *PsEND1::barnase-barstar* “Hillary” ablated anther showing necrotic tissues and a short filament. **(N)** *Pelargonium zonale* stamens from WT flowers 1 day prior to anthesis showing fully developed locules and filaments. **(O)** *P. zonale* transgenic *PsEND1*::*barnase-barstar* stamens showing collapsed and necrotic anthers at the end of a short filament instead of a normal four-lobed anther with a fully expanded filament. **(P)** *A. thaliana* WT plant (left) showing fruits (siliques, white arrow) after flower fertilization compared with a more branched transgenic male sterile *PsEND1::barnase-bastar* plant showing more branches and flowers and the absence of siliques (right). **(Q)** Comparative panel showing how the flowering branches of WT tobacco plants were fertilized normally and produced capsules (left arrowhead), while the branches of transgenic plants do not show the formation of capsules and continue growing to produce more unfertilized flowers, which finally senesce (right arrowhead). **(R)** WT Micro-Tom tomato fruit showing the presence of seeds (upper arrowhead) compared with a seedless *PsEND1*::*barnase-barstar* parthenocarpic fruit (bottom arrowhead). Scale bars represent 2.0 mm in **A** and **B**; 100 μm in **A** and **B** center; 200 μm in **A** and **B** right; 0.5 cm in **C**, **D**, **E**, **F**, **G**, and **H**; 0.2 cm in **I**, **J**, **N**, and **O**; 400 μm in **K**, **L**, and **M**; 5.0 cm in **P** and **Q**; and 1.0 cm in **R**. Adapted from Roque et al. (2007), García-Sogo et al. (2010, 2012), Beltrán et al. (2007), and Medina et al. (2013).

The *PsEND1*::*barnase-barstar* chimeric construct provided efficient male sterility by early anther ablation in two Brassicaceae: *Arabidopsis thaliana* and *Brassica napus* (Roque et al., 2007). *A. thaliana* plants were transformed by floral dip. The anther development was arrested in the transgenic plants at early stages and hook-shaped structures at the end of a short filament were formed instead of normal locules (Figures 2A,B). The formation of short filaments is commonly associated with male sterility or reduced fertility as a consequence of incomplete anther development (Mariani et al., 1990). All the transgenic lines obtained failed to produce siliques and seeds. Transgenic *Arabidopsis* plants harboring only the *PsEND1::barstar* chimeric gene were also generated to check the reversibility of the system to restore fertility. After crossing with the male sterile plants previously generated, fertile plants showing restored anthers were obtained (Roque et al., 2007).

New hybrid plant varieties with increased yield have been obtained by breeders in the last decades. Hybridization of self-pollinating crops (e.g., oilseed rape and tomato) has been performed traditionally by manual emasculation followed by fertilization with pollen of the selected donor. Nevertheless, this practice is a tedious and time-consuming process and full sterility is not guaranteed. Therefore, engineered male sterility is a suitable alternative to prevent self-pollination in both plant species.

Oilseed rape is a 30% allogamous and a 70% autogamous, thus to produce hybrid lines it is necessary the implementation of an efficient system for the control of pollination. For this purpose, we genetically transformed *B. napus* cv. Drakkar plants with the *PsEND1::barnase-barstar* construct to find out whether the pea *PsEND1* promoter could be functional and produce male sterility in a distantly related crop (Roque et al., 2007). Primary transformants showed collapsed anthers with short filaments (Figures 2C,D). The absence of pollen grains into the transgenic locules was confirmed by light microscopy. The unpollinated transgenic carpels do not produced fruit and seeds, while the carpels of untransformed control plants were fertilized and formed normal fruits and seeds.

The *PsEND1::barnase-barstar* construct also resulted in efficient male sterility in two Solanaceae: *Nicotiana tabacum* and *Solanum lycopersicum* (Roque et al., 2007). In transgenic *N. tabacum* plants, the flowers presented collapsed anthers (arrowhead shape) with no pollen grains at the end of a short filament (Figures 2E,F).

Tomato is a widespread crop all over the world and different systems have been developed to generate male sterility in this crop. However, these systems are not very useful at the commercial level due to the difficulties to maintain pure male sterile lines. The *PsEND1::barnase-barstar* construct also showed high efficiency in the generation of male sterile lines of two tomato cultivars: Micro-Tom and Moneymaker (Roque et al., 2007; Medina et al., 2013). In comparison with the non-transformed control plants, the flowers were male sterile, showing collapsed anthers with necrotic tissues and without pollen grains (Figures 2G,H). Unlike in the wild-type flowers, the carpel was not covered by the anthers forming the staminal cone. The ploidy level of all the tomato transgenic lines obtained was checked and only the diploid ones were retained to avoid misleading results. Backcrosses of all the transformed lines using pollen from wild-type (WT) plants produced normal tomato plants harboring fruits with seeds, indicating that female fertility was not affected in the transgenic *PsEND1::barnase-barstar* plants. Segregation analyses indicated that, in the next generation, the inheritance and stability of the incorporated transgenes were fully conserved.

## Generation of Non-Allergenic Pollen-Free Ornamental Plants Using the *PsEND1*::*Barnase* System

In the last decades, conventional breeding has been extensively used to introduce commercially interesting traits into different ornamental plants. At present, genetic engineering allows specific modifications of single traits, with potential interest for consumers, in already successful commercial varieties. Allergic responses to the pollen of several ornamental species have high incidence in the general atopic population and especially among gardeners and flower growers (Goldberg et al., 1998).

The *PsEND1::barnase-barstar* construct has been assayed in two of the most grown flowering plants in Europe: *Kalanchoe* and *Pelargonium*. In the last years, different traits of interest have been introduced into *Kalanchoe blossfeldiana* by genetic engineering, leading to the generation of dwarf genotypes, new floral colors, more compact phenotypes, root inducing (Ri)-lines, reduced sensitivity to ethylene, and marker-free transgenic varieties (Christensen et al., 2008; Sanikhani et al., 2008; Topp et al., 2008; Thirukkumaran et al., 2009). Therefore, the implementation in this ornamental species of a reliable and efficient male sterility system would be of interest to avoid allergic responses of the potential consumers and to produce environmentally friendly plants by preventing gene flow between the existing genetically modified cultivars and related species.

Transgenic lines of two *K. blossfeldiana* cultivars (“Hillary” and “Tenorio”) carrying the construct *PsEND1*::*barnase-barstar* were generated from leaf explants (García-Sogo et al., 2010). Transgenic “Tenorio” and “Hillary” stamens, compared with the non-transformed ones, showed dramatic differences in development (Figures 2I–M). In WT flowers at 1 day prior to anthesis, the anthers showed four locules with viable pollen grains (Figure 2K), while in the transgenic ones the anthers were replaced by collapsed and necrotic structures without pollen grains located at the end of a short filament (Figures 2L,M).

Similarly, engineered *PsEND1::barnase-barstar Pelargonium zonale and P. peltatum* (García-Sogo et al., 2012) male sterile flowers showed collapsed and necrotic anthers without pollen grains at the end of a short filament (Figures 2N,O). Cross-pollination of the *Kalanchoe* and *Pelargonium* male sterile lines using WT pollen resulted in the production of normal fruits and seeds, indicating that female fertility was not affected in the transgenic plants. Segregation studies in both transgenic plants indicated that the inheritance and stability of the transgenes were maintained in the progeny.

## Side Effects in the *PsEND1*::*Barnase-Barstar* Male Sterile Plants With Interesting Biotechnological Applications

It has been observed in different *PsEND1*::*barnase-barstar* male sterile plants some interesting side effects that could be of interest to be exploited from a biotechnological point of view. We observed increased plant longevity, branching, and number of flowers that suggest the redirection of resources usually directed to the production of fruits and seeds. The scientific explanation of these phenomena could be related with a sink’s matter. Engineered male sterile plants use the sucrose that has not been used in the formation of fruits and seeds in the production of more branches and flowers and the final consequence is the prolongation of the plant’s life (Beltrán et al., 2007).

In the *PsEND1*::*barnase-barstar* male sterile plants of *Arabidopsis* and tobacco we have observed increased branching and flower number, leading to a drastic change in plant architecture (Beltrán et al., 2007). The *Arabidopsis* control plants begin the senescence process after production of fruits. However, male sterile plants do not develop siliques but produce more branches of first, second, third, and fourth order in the axillary nodes of both rosetta and cauline leaves. These branches also develop more flowers than WT plants (Figure 2P). Similarly, tobacco *PsEND1*::*barnas*e-*barstar* plants continue producing flowers after WT plants finished the production of capsules and begin the senescence process (Figure 2Q). Both *Arabidopsis* and tobacco male sterile plants showed increased plant longevity, producing more branches and flowers. Therefore, our engineered male sterility system could be useful to reduce the need for deadheading to extend the flowering period or the invasive potential of some ornamental species, and also to increase biomass production in forest trees (Jain and Minocha, 2000).

In the engineered male sterile tomato plants, we have observed the production of seedless parthenocarpic fruits as a consequence of the early anther ablation (Roque et al., 2007; Medina et al., 2013; Rojas-Gracia et al., 2017). Tomato fruit set and development are strongly affected by changes in the environmental conditions, thus autonomous fruit set independent of fertilization is a desirable trait in this crop species. We generated *PsEND1*::*barnase-barstar* male sterile transgenic plants producing parthenocarpic fruits in two tomato cultivars: Micro-Tom and Moneymaker. The ovaries of these plants were able to grow in the absence of fertilization and subsequently producing parthenocarpic fruits (Figure 2R). In this process, early ablation of the anthers is essential to activate the developing of the transgenic ovaries into seedless fruits, in the absence of signals produced during pollination and fertilization. *PsEND1*::*barnase-barstar* tomato plants of the commercial cultivar Moneymaker showed that the parthenocarpic development of the fruit is not detrimental to fruit quality. Several elite lines were identified and selected for their increased yield and quality performance. In fact, the changes detected in the metabolic profile of the ripe fruits from these lines indicated an improved organoleptic and nutritional quality. In addition, these male sterile plants could be used in hybrid breeding applications as very convenient parental lines. The transgenic lines generated could also be useful tools to investigate the molecular mechanisms accountable for the observed metabolic phenotypes, and also to understand the connection between impaired anther development and parthenocarpy (Medina et al., 2013; Rojas-Gracia et al., 2017, 2019).

## Conclusion and Perspectives

Future advances in crop species to produce more feed and food contributing to a sustainable agriculture will require synergy among several research fields, including traditional breeding, crop management, physiology, genetics, and biotechnology (Beltrán and Cañas, 2018). Natural male-sterile mutants have appeared in the germplasm of the more used cultivars; however, their economic value was not recognized and they were less in few generations. However, after the concept of heterosis (Shull, 1908), the benefits on the use of male sterility in hybrid seed production to increase crops yield were appreciated. Male sterility could be obtained by different failures in microsporogenesis, release of pollen grains, or pollen germination that do not affect the female reproductive system; therefore, the male sterile plants can produce viable seeds after manual pollination.

Engineered male sterility can be achieved by using anther- or pollen-specific promoters fused to a ribonuclease gene to produce ablation of specific cell types that are essential for proper anther development. The use of new anther-specific promoters showing very early expression, such as the *PsEND1* promoter, could help to produce new high-yielding hybrid cultivars and environmentally friendly GM crops by preventing gene flow between genetically modified plants and compatible species. We have developed a simple and reliable system to produce engineered nuclear male sterile plants using the pea *PsEND1* promoter, which specifically direct the expression of the *barnase* gene to different anther tissues involved in anther architecture in all plant species tested. The *PsEND1* promoter is currently used by different research groups in a wide range of plant species to produce male sterility, including forest trees.

In legumes, the obtaining of new hybrid cultivars will contribute to enhance yield and productivity by exploiting the hybrid vigor generated. Cytoplasmic nuclear male sterility has been widely used by breeders to achieve breakthroughs in the productivity of several crops, including legumes, generating hybrid lines. Among the high-protein legumes, the first high-yielding hybrid of pigeon pea, based in cytoplasmic nuclear male sterility and partial natural outcrossing, was recently released in India with record 3–4 t/ha of grain yield and with 30–40% yield advantage over 3 years of testing in farmers’ fields. Also, under high-input conditions and good management yields, up to 4,000–5,000 kg/ha have been recorded by farmers (Saxena et al., 2013; Saxena and Hingane, 2015).

The genetically engineered male sterility approach described here, which uses an anther-specific promoter from a legume, provides new opportunities to the breeders for enforcing pollination control in hybrid seed production systems and might help to produce new hybrid cultivars in different legume species.

## Author Contributions

ER, RH, and CG-M performed the experiments. CG-M, JB, and LC conceived the experiments, analyzed the data, and wrote the grants that funded this work. LC wrote the manuscript. All authors read and approved the final version of this manuscript.

## Conflict of Interest Statement

The authors declare that the research was conducted in the absence of any commercial or financial relationships that could be construed as a potential conflict of interest.
